# High-Intensity Interval Cycle Ergometer Training in Parkinson's Disease: Protocol for Identifying Individual Response Patterns Using a Single-Subject Research Design

**DOI:** 10.3389/fneur.2020.569880

**Published:** 2020-10-22

**Authors:** Erwin E. H. van Wegen, Mark A. Hirsch, Wilma D. J. van de Berg, Chris Vriend, Marc B. Rietberg, Mark A. Newman, Tim Vanbellingen, Odile A. van den Heuvel

**Affiliations:** ^1^Department of Rehabilitation Medicine, Amsterdam Movement Sciences, Amsterdam Neuroscience, Amsterdam University Medical Center (UMC), Vrije Universiteit Amsterdam, Amsterdam, Netherlands; ^2^Department of Physical Medicine and Rehabilitation, Carolinas Medical Center, Carolinas Rehabilitation, Charlotte, NC, United States; ^3^Department of Anatomy & Neurosciences, Amsterdam Neuroscience, Amsterdam University Medical Center (UMC), Vrije Universiteit Amsterdam, Amsterdam, Netherlands; ^4^Department of Psychiatry, Amsterdam Neuroscience, Amsterdam University Medical Center (UMC), Vrije Universiteit Amsterdam, Amsterdam, Netherlands; ^5^Department of Rehabilitation Medicines, Amsterdam Movement Sciences, Amsterdam University Medical Center (UMC), Vrije Universiteit Amsterdam, Amsterdam, Netherlands; ^6^Neurocenter, Luzerner Kantonsspital, Lucerne, Switzerland; ^7^Gerontechnology and Rehabilitation Group, University of Bern, Bern, Switzerland

**Keywords:** Parkinson's disease, high intensity (strenuous) exercise, neuroplastic changes, BDNF, NFL, synuclein alpha

## Abstract

**Background:** People with Parkinson's disease (PD) experience not only motor problems but also non-motor problems that seriously impede their daily functioning and quality of life. The current pharmacologic treatment of PD is symptomatic, and alternative rehabilitation treatments, which preferably also have a disease-modifying effect and promote neuroplasticity, are needed. Recent studies suggest that high-intensity interval training (HIIT) is promising for promoting neuroplasticity in human PD, with short training time and reduced burden. Biomarkers for neuroplasticity such as brain-derived neurotrophic factor (BDNF) and neurodegeneration (including neurofilament NfL and α-synuclein) may play a role, but their response to HIIT is not well-investigated.

**Objectives:** The aims of this study were (1) to study the effects of 4 weeks of HIIT compared with 4 weeks of continuous aerobic exercise on motor and non-motor outcomes of PD and (2) to investigate the association between HIIT, motor/non-motor performances changes, and blood biomarker levels for neuroplasticity and neurodegeneration.

**Study Design:** Single-subject research design with alternating treatment setup (ABACA) and frequent repeated measurements was used. Each participant received different intervention conditions (B/C) interspersed with baseline periods (A, i.e., ABACA or ACABA), and frequent repeated assessment of outcome measures is done to quantify within-subject, individual response patterns with sufficient power for data analysis. Blood samples were collected once a week in the baseline and training phases (A1 and B/C) and once every 2 weeks in the washout phases (A2 and A3).

**Intervention:** Four subjects with PD on stable dopaminergic medication, two in Hoehn–Yahr stage 1–2, and two in Hoehn–Yahr stage 2.5–3 followed an ABACA or ACABA schedule, consisting of blocks with 30-min sessions of “B” (HIIT) or 50-min sessions of “C” [continuous aerobic exercise (CAE)] 3×/week for 4 weeks, separated by baseline “A” periods of 8 weeks for a total duration of 28 weeks.

**Outcome Measures:** Outcome measures include disease status [Movement Disorder Society-sponsored revision of the Unified Parkinson's Disease Rating Scale (MDS-UPDRS)], blood biomarkers (BDNF, Nfl, and α-synuclein), measures for functional mobility (including an activity tracker), and activities of daily living, as well as cognition, mood, biorhythm (sleeping problems), and quality of life.

**Data Analysis:** Visual analysis of trends in level, slope, and variability in response patterns was carried out, confirmed by longitudinal regression analysis with phase (ABACA) as the independent variable.

## Introduction

Parkinson's disease (PD) is a complex, chronic, highly disabling progressive neurodegenerative movement disorder, characterized by the loss of dopamine neurons in the substantia nigra (1). The disease is characterized by motor (e.g., tremor, gait disturbance, bradykinesia, and falls) and also non-motor (e.g., depression, reduced cognitive performance, and biorhythm/sleep) symptoms (2). To date, there is no cure. Current treatment paradigms are mainly aimed at alleviating motor and psychiatric features but do not consider individual differences in treatment response or molecular profile. Moreover, several motor (e.g., gait disturbances and balance disturbances) and non-motor (e.g., mood disturbances, cognitive functioning, and sleep problems) features respond insufficiently to current pharmacological treatments; and unfortunately, we do not have rehabilitative strategies that halt or slow down disease progression (2, 3). Frequent physical exercise training is increasingly recognized as an effective therapy for PD and other neurodegenerative diseases that may even exert disease-modifying influences through neuroplastic mechanisms (4, 5).

Aerobic and/or progressive resistance exercise based on the American College of Sports Medicine recommendation of 3×/week, 30–60 min/session for at least 12 weeks demonstrate long-term (>12 weeks) improvements in both motor performance [e.g., walking speed, aerobic capacity, and lower-limb muscle strength; (6)] and cognitive performance (attention and executive functioning) in PD patients (3, 7). Shorter-lasting aerobic exercise programs (3×/week, 30–60 min/session for 4 weeks) may also show improvements in motor performance (e.g., walking speed and aerobic capacity), though the long-term effects of short-term aerobic exercise are less consistent (4).

Physical training studies show promising results in terms of exercise-induced neuroplasticity, at both symptom and molecular levels in human and animal models of exercise and PD (8). For example, voluntary treadmill running in mice for 40 min/day, 5 days/week for 18 weeks improved motor coordination and aerobic capacity and reduced nigrostriatal neuronal loss (9). The improvements were associated with increased levels of BDNF in the substantia nigra and striatum. A recent review and a meta-analysis showed that HIIT has acute and long-term effects on serum BDNF levels (10, 11). Several studies suggest that serum BDNF concentration levels are reduced in PD patients [for example, Frazzitta et al. (12)]. Low BDNF is associated not only with cognitive impairments in PD patients (13) but also with mood disturbances (14). Biomarkers for neuroplasticity, such as BDNF, and biomarkers for (synaptic) neurodegeneration (including neurofilament NfL and α-synuclein) are, respectively, decreased and increased in PD patients compared with age-matched controls (15–17). Studies suggest intensive physical training to be promising for modulating these biomarkers in people with PD (11, 12). BDNF has shown to respond well to intensive physical training; for serum NfL and α-synuclein, this has not yet been properly investigated.

A promising, time-efficient, and highly effective physical exercise strategy is “high-intensity interval training” (HIIT), which only recently is being explored in clinical research (18). HIIT encompasses several intervals of short-lasting, high-intensity bouts alternated with low-intensity bouts, that has shown to match or even surpass the cardiovascular responses to moderate continuous aerobic exercise (CAE) (19). This increased efficiency is achieved in a shorter period of total training time, which may enhance the motivation of PD patients and help promote adopting a more active lifestyle (19). For example, HIIT for three 40-min sessions per week for 8 weeks has been suggested to evoke enhanced adaptive aerobic capacity (e.g., increased aerobic capacity) as well as increased cognitive performance, as compared with CAE in healthy older adults and older adults with mild cognitive impairment (19). Similar results have been found for adult type 2 diabetes mellitus patients (18) and adult cardiovascular patients (4).

Only a few studies to date have investigated the effect of HIIT on motor and non-motor outcomes in PD. High feasibility of HIIT in PD was recently demonstrated (20): participants were able to be complete 45 min of HIIT three times a week for 12 weeks without intervention-related dropouts and >80% compliance. Group session HIIT consisted of an exercise gym circuit designed to elicit a physiological response indicative of high-intensity exercise (≥85% HRmax). Fernandes et al. (21) performed a pilot randomized controlled single-blind trial to study the effect of 12 weeks of HIIT vs. moderate-intensity continuous exercise (MICE) training in PD. They enrolled 20 participants, with Hoehn–Yahr stage 1–3, who performed walking/jogging training three times per week for 12 weeks. The HIIT protocol consisted of a 1-min walking/jogging bout at rating of perceived exertion (Borg scale RPE) scale 15–17 level alternated with 2 min of walking at 9–11 level of RPE during a 25-min session. MICE training consisted of 26 min of walking/jogging at 11–14 level of RPE. HIIT improved 6-min walk test distance and increased endothelial reactivity (a marker for increased blood flow). These values did not change with MICE. They did not measure non-motor outcomes. O'Callaghan et al. (22) entered the sample by Harvey et al. into a new comparison in a 12-week pilot study of HIIT or MICE. Their study supports the feasibility of HIIT in PD and found that HIIT increased circulating serum brain-derived neurotrophic factor (BDNF), a protein that may play a key role in neuroplasticity maintaining or improving brain functions (10). BDNF levels were higher after HIIT compared with continuous moderate-intensity aerobic training (22).

The combined evidence of animal and human studies suggests that motor performance, cognitive performance, and mood in PD are related and that biomarkers for neuroplasticity and neurodegeneration are associated with both motor and non-motor features. This underpins the importance of disease-modifying treatment in PD including aerobic and/or progressive resistance exercise as adjuncts to pharmacological treatment for the control of motor and non-motor features. As such, determining the optimal type and dosage of exercise interventions to obtain these neuroplastic effects is an important focus of current neurorehabilitation research in PD (8, 23).

Researchers argue that the heterogeneity of patient populations and use of classic intervention study design are major confounding factors in making progress toward determining optimal type and dosage of exercise interventions (24). Within-participant research designs with frequently repeated measurements (i.e., single case experimental designs) may be more suitable than group studies to capture individual recovery patterns. This design allows for more continuous repeated measures of the target outcomes, at baseline, during, and after the intervention period (25). Incorporation of many repeated measures allows a detailed analysis of individual responses with adequate power. No single study has compared the effects of HIIT with CAE on changes in motor performance, cognitive abilities, mood disturbances, and biorhythms/sleep in people with PD and whether improvements are associated with quantitative changes in blood-based biomarkers (BDNF, NfL, and α-synuclein).

Therefore, the first objective of this pilot study is to explore the response patterns of motor and non-motor performance and serum biomarker levels to 4 weeks of HIIT compared with 4 weeks of traditional moderate CAE in four single subjects with PD. The second objective is to explore the associations between changes in motor and non-motor outcome measures and serum neuroplasticity and neurodegeneration biomarkers for both exercise conditions.

Our central hypothesis is that HIIT is more effective at improving motor performance, cognition, mood, and sleep as well as increasing concentration of BDNF and decreasing NfL and α-synuclein levels than moderate-intensity CAE. In addition, we expect that HIIT-induced changes in serum BDNF, NfL, and α-synuclein protein levels are associated with changes in motor and non-motor performance of PD patients.

## Methods

### Study Design

We will use a single-subject alternating treatment approach with A1-B-A2-C-A3 or A1-C-A2-B-A3 design to study the effects of HIIT on motor and non-motor performance and neuroplasticity and neurodegeneration biomarkers such that subjects will perform both the HIIT (B-phase) and the moderate CAE training (C-phase) for 4 weeks. After a baseline (A1) period of 4 weeks, the first intervention period of 4 weeks will take place (B or C), followed by a washout period of 8 weeks without an intervention (A2), the second intervention period of 4 weeks (B or C), and finally a third washout period of 8 weeks (A3). Each patient therefore experiences the full set of treatment approaches. The order of interventions B and C will be randomized for each participant (i.e., ABACA or ACABA, two of each, four participants total) using a random sequence generator. Assessment will be done by Good Clinical Practice (GCP)-trained blinded assessors.

### Study Population

This exploratory pilot study aims to recruit a convenience sample of four subjects with confirmed idiopathic PD on stable dopaminergic medication via the outpatient clinic for movement disorders at Amsterdam UMC, Vrije Universiteit Medical Center, Amsterdam, the Netherlands. Patients are asked in the study information letter to maintain a stable medication schedule throughout the study, unless the treating neurologist deems it medically necessary to adapt drug treatment. When applicable, this will be recorded and taken into account in the analysis. The study was approved by the Medical Ethics Committee of Amsterdam UMC, Vrije Universiteit Medical Center (nr. 2029.083). The study will be conducted in compliance with the Declaration of Helsinki.

### Inclusion and Exclusion Criteria

Inclusion criteria are (1) diagnosis of idiopathic PD according to UK Brain Bank criteria (26); (2) Hoehn–Yahr stage 1 (*n* = 2 patients) and 2.5–3 (*n* = 2 patients) as confirmed by a trained assessor; (3) age between 55 and 80 years; (4) sufficient cognition to comprehend training instruction [Montreal Cognitive Assessment (MoCA) score > 21]; and (5) able to provide written informed consent. Exclusion criteria are (1) history of neurologic deficits other than PD; (2) severe fluctuating responses to dopaminergic medication; (3) psychiatric, musculoskeletal, or metabolic disorders prohibiting participation in intensive exercise training; (4) cardiovascular disorders or cardiac risk prohibiting participation in intensive exercise training as assessed by Lausanne Questionnaire [>2 risk items scored “yes”; Bille et al. (27)]; and (5) participation in a professionally supervised high-intensity therapy/exercise program to improve fitness in the 2 months before inclusion.

### Measurement Procedures and Training

All testing and training will be performed in the “on” state, ~1–1.5 h after medication intake. Prior to allocation, subjects will complete baseline testing for demographics and for motor and non-motor symptoms (Table 1). Assessment of demographics and baseline characteristics will include, age, height, weight, sex, disease duration, Movement Disorder Society-sponsored revision of the Unified Parkinson's Disease Rating Scale (MDS-UPDRS I to IV), Hoehn–Yahr stage and LEDD (levodopa equivalent daily dose), medication schedule and usage (dopaminergic, psychopharmacological, and any other medications), and number of falls in past month.

Table 1Assessment schedule for outcomes across the 28-week study period.

**Table:** 

**Schedule**																									
	**Phase**	**Baseline A1**												**Intervention B/C**											
	**Week**	**1**			**2**			**3**			**4**			**1**			**2**			**3**			**4**		
	**Session**	**s1**	**s2**	**s3**	**s1**	**s2**	**s3**	**s1**	**s2**	**s3**	**s1**	**s2**	**s3**	**s1**	**s2**	**s3**	**s1**	**s2**	**s3**	**s1**	**s2**	**s3**	**s1**	**s2**	**s3**
Tests	Cardiac Screening	x																							
	Demographics	x																							
	VO_2_ test												x												
Motor	UPDRSI-IV	x												x											
	TUG	x			x			x			x			x											
	10m walk	x			x			x			x			x			x			x			x		
	one-leg-stance	x			x			x			x			x			x			x			x		
Cognition	MoCa	x																							
	Stroop test	x												x											
	TMT	x												x											
	PD-CFRS	x												x											
	VAS-concentration	x			x			x			x			x	x	x	x	x	x	x	x	x	x	x	x
Mood	BDI	x												x											
	VAS-mood	x			x			x			x			x	x	x	x	x	x	x	x	x	x	x	x
Sleep	Scopa sleep	x												x											
	VAS-sleep	x			x			x			x			x	x	x	x	x	x	x	x	x	x	x	x
ADL	NEAI	x												x											
QoL	PDQ-8	x												x											
Blood	BDNF/NfL, α-syn	x			x			x			x			x			x			x			x

**Table:** 

	**Phase**	**Baseline A2**																								**Intervention C/B**											
	**Week**	**1**			**2**			**3**			**4**			**5**			**6**			**7**			**8**			**1**			**2**			**3**			**4**		
	**Session**	**s1**	**s2**	**s3**	**s1**	**s2**	**s3**	**s1**	**s2**	**s3**	**s1**	**s2**	**s3**	**s1**	**s2**	**s3**	**s1**	**s2**	**s3**	**s1**	**s2**	**s3**	**s1**	**s2**	**s3**	**s1**	**s2**	**s3**	**s1**	**s2**	**s3**	**s1**	**s2**	**s3**	**s1**	**s2**	**s3**
Tests	Cardiac Screening																																				
	Demographics																																				
	VO_2_ test																								x												
Motor	UPDRSI-IV	x																								x											
	TUG	x						x						x						x						x											
	10m walk	x						x						x						x						x			x			x			x		
	one-leg-stance	x						x						x						x						x			x			x			x		
Cognition	MoCa																									x											
	Stroop test	x																								x											
	TMT	x																								x											
	PD-CFRS	x																								x											
	VAS-concentration	x			x			x			x			x			x			x			x			x	x	x	x	x	x	x	x	x	x	x	x
Mood	BDI	x																								x											
	VAS-mood	x			x			x			x			x			x			x			x			x	x	x	x	x	x	x	x	x	x	x	x
Sleep	Scopa Sleep	x																								x											
	VAS-sleep	x			x			x			x			x			x			x			x			x	x	x	x	x	x	x	x	x	x	x	x
ADL	NEAI	x												x												x											
QoL	PDQ-8	x												x												x											
Blood	BDNF/NfL, α-syn	x						x						x						x						x			x			x			x

**Table:** 

	**Phase**	**Baseline A3**																																			
	**Week**	**1**			**2**			**3**			**4**			**5**			**6**			**7**			**8**		
	**Session**	**s1**	**s2**	**s3**	**s1**	**s2**	**s3**	**s1**	**s2**	**s3**	**s1**	**s2**	**s3**	**s1**	**s2**	**s3**	**s1**	**s2**	**s3**	**s1**	**s2**	**s3**	**s1**	**s2**	**s3**
Tests	Cardiac Screening																								
	Demographics																								
	VO_2_ test																								
Motor	UPDRSI-IV	x																							x
	TUG	x						x						x						x					x
	10m walk	x						x						x						x					x
	one-leg-stance	x						x						x						x					x
Cognition	MoCa																								x
	Stroop test	x																							x
	TMT	x																							x
	PD-CFRS	x																							x
	VAS-concentration	x			x			x			x			x			x			x					x
Mood	BDI	x																							x
	VAS-mood	x			x			x			x			x			x			x					x
Sleep	Scopa Sleep	x																							x
	VAS-sleep	x			x			x			x			x			x			x					x
ADL	NEAI	x												x											x
QoL	PDQ-8	x												x											x
Blood	BDNF/NfL, α-syn	x						x						x						x					x

*Planning of baseline (A1, A2, and A3), intervention (B and C), and measurements (crosses indicate time movements). Pre–post phase measurements will be performed before and after both baseline and both intervention phases*.

*UPDRS, MDS-Unified Parkinson's Disease Rating Scale; MoCa, Montreal Cognitive Assessment; PD-CFRS, PD Cognitive Functioning Rating Scale; Stroop, Stroop test; VAS, visual analog scale; BDI, Beck Depression Inventory; ADL, activities of daily living; Scopa Sleep, Sleep scale from Scopa; NEAI, Nottingham Extended ADL index; QoL, quality of Life; PDQ-8, Parkinson's Disease Questionnaire; BDNF, brain-derived neurotrophic factor; NfL, neurofilament light; α-syn, alpha synuclein*.

#### VO_2_-Max Test Procedure

At the end of the A1 phase, subjects will perform a VO_2_-Max test to determine individual training load, as described by Mavromatti et al. (28). In brief, the exercise test will be conducted on an electronically braked cycle ergometer (Excalibur Sport, Lode, the Netherlands), integrated with a cardiopulmonary-monitoring system that controls the work rate protocol on the ergometer and records breath-by-breath measurements of VO_2_, VCO_2_, ventilation, and heart rate (Cosmed Benelux BV, the Netherlands) throughout the test. Handlebars and saddle of the cycle ergometer will be adjusted to match each participant's anthropometrics. The work rate protocol consists of 2-min steps starting with unloaded cycling and then increasing to 50 W, and thereafter increasing in steps of 25 W. While the ergometer maintains a constant workload, independent of cadence, participants are instructed to aim for cadence of ~60–80 rpm. At the end of each step, participants are asked to rate their level of exertion (rating of perceive exertion) using the Borg RPE 10-point category ratio scale (Borg CR10 scale, “0” to “10”) (29). Participants are verbally encouraged to carry on for as long as possible. The exercise test is terminated by voluntary exhaustion, by a cadence <45 rpm or for safety reasons, compliant with the American College of Sports Medicine's guidelines for clinical exercise testing (30). Subjects are asked to abstain from exercise and consumption of alcohol or caffeine 12 h prior to testing.

#### Outcome Measures

Outcome measures are chosen based on recommendations by the Movement Disorders Society (31), the 2014 European guideline of physical therapy for PD (32), and recommendations by the members of the project team, including several PD patients. Table 1 shows the timeline and assessment schedule for demographics and all outcome parameters. Demographics include age, sex, disease duration, Hoehn–Yahr stage, medication schedule and dosage, and MoCA score.

The primary study parameter is disease severity measured every 4 weeks during baseline A1 and A2 and intervention and two times during baseline A3 with the MDS-UPDRS part III (motor examination) during on-state (33).

Secondary outcome parameters were pre–post intervention outcome measures will be administered before and after each study phase; and disease status with MDS-UPDRS 1, II, and IV (33). Cognition will be evaluated with the MoCA (34), the Stroop test (35), and the Trail Making Test for executive function [TMT; Olchik et al. (36)] and the PD Cognitive Functioning Rating Scale (PD-CFRS) (37). Activities of daily living and quality of life were assessed with self-report questionnaires: the Nottingham Extended ADL index (NEAI) (38) and the Parkinson Disease Questionnaire (PDQ-8) (39). Mood and sleep will be assessed using the Beck Depression Inventory questionnaire (31) and the Scopa Sleep scale (40), respectively.

Weekly outcomes are motor performance, namely, the 10-meter walk test (41), the Timed “Up-and-Go” test (TUG) (42), and the one-leg stance test (43). In addition, daily physical activity with a wrist-worn activity tracker will be recorded throughout the study. For weekly self-report outcomes, we will use Ecological Momentary Assessment methodology (44), asking frequent (weekly in baseline phases, three times a week in intervention phases), short online survey questions about non-motor functions using Castor EDC (45): Mood will be assessed with a visual analog scale (VAS) score rating: “Can you rate your current mood on a scale from “1” to “10”?” (a score of “1” represents “very sad”; a score of “10” represents “very happy”). Sleep performance will be assessed with a VAS score rating: “Can you rate how well you slept last night on a scale from “1” to “10”?” (A score of “1” represents “very bad”; a score of “10” represents “very good”). Cognitive function will be assessed with a VAS score for “ability to concentrate”: “Can you rate well you are able to concentrate on daily tasks on a scale from “1” to “10”?” (a score of “1” represents “very bad”; a score of “10” represents “very good”). In addition, we will ask the subjects to keep a logbook of their attended sessions and perceived effort (RPE).

Blood samples (for serum BDNF, NfL, and α-synuclein) will be collected by a trained nurse once a week in the baseline and training phases (A1 and B/C). Blood draw frequency is decreased to once every 2 weeks in the washout phases (A2 and A3) to reduce patient burden. Twenty-milliliter blood samples will be taken from the antecubital vein at rest in the morning hours between 08:00 and 10:00 in the fasting state. We will use anticoagulant-free tubes, which be placed on ice (−80°C) until further analysis. BDNF concentration will be measured with a commercially available and in-house validated kit (Quanterix Simoa). Serum NfL and α-synuclein concentrations will be measured using an in-house developed Homebrew Simoa assay (17, 46).

### Training Protocol

**Figure 1:**
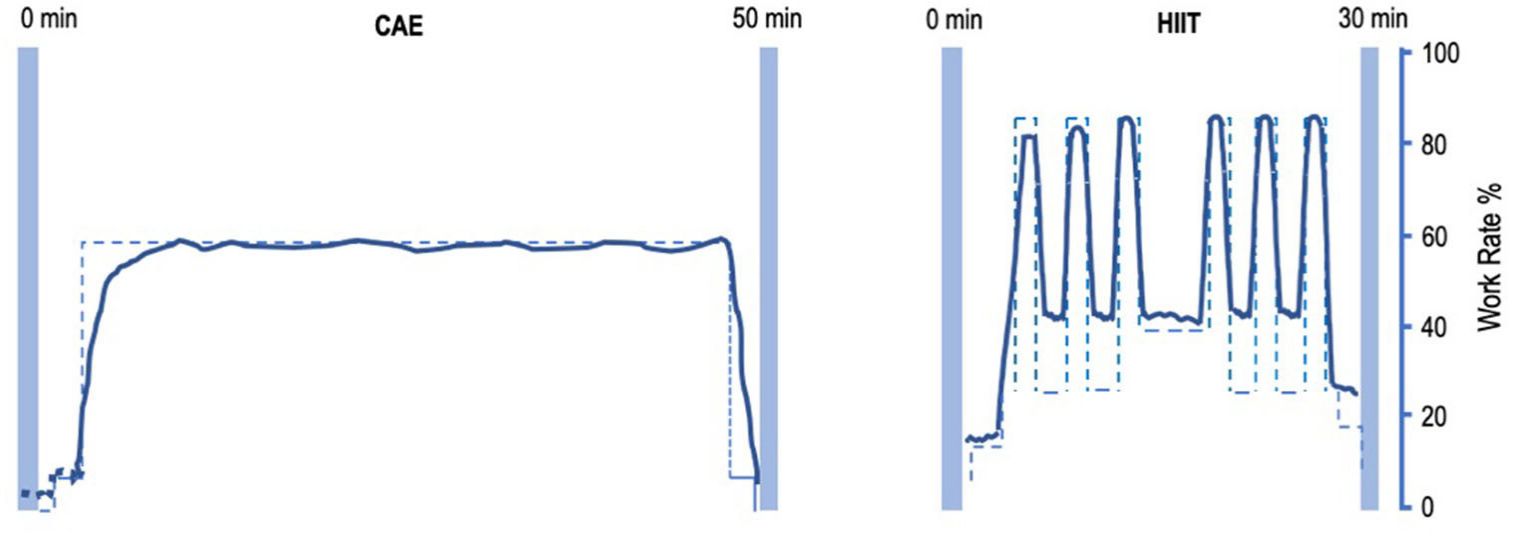
Intensity-work rate plot for CAE and HIIT session. **Left:** intensity-work rate plot for a 50-min CAE intervention session. **Right:** intensity-work rate plot for a 30-min HIIT intervention session. CAE training intensity will be at about 55% W-max, HIIT will alternate between training intensity 35% W-max during low-intensity intervals and >85% W-max at high-intensity intervals. CAE, continuous aerobic exercise; HIIT, high-intensity interval training.

Subjects will perform HIIT or moderate CAE on a stationary bicycle (Lode Corival, Lode Inc.) 3×/week for two periods of 4 weeks in a group of four subjects (Figure 1). The order of the intervention phases will be randomized. Training will take place on Mondays, Wednesdays, and Fridays, to allow for sufficient recovery periods between training sessions.

Each HIIT session is about 30 min of interval training, starting with a 5 min of warm-up at 25–35% W-max. Subsequently, six to eight blocks of HIIT will be performed, alternating 45 s at >85% VO_2_-max, with 90 s at 30–40% W-max. After the interval block, 5 min of cooling-down will be performed at 20–35% W-max. Workload will be progressively adapted using a fixed weekly schedule or upon therapist decision by adding intervals at the end of the sessions.

Each CAE session is about 50 min of continuous training at a preset workload of about 55% W-max. Workload will be progressively adapted after every week by reducing rest time, based on a fixed schedule or upon therapist decision. A trained physiotherapist-supervisor, assisted by GCP-trained interns from the Faculty of Human Movement Sciences will assist the subjects with water, music, and motivational encouragements. Throughout the study, patients are asked to maintain their regular medication.

The Ecological Momentary Assessments taken weekly (A phases) or every other day (B/C phases) using the online survey throughout the study period will act automatically as reminders and will help with compliance to training and assessments. We will comply with consensus recommendations of Slade et al. (47) regarding reporting on exercise interventions.

### Statistical Analysis

Although the protocol specifies primary and secondary study outcomes, we will assess the results based on a combination of those parameters.

For example, the primary research parameter that we have specified is the MDS-UPDRS-III, which is measured before and after each phase. If, under the influence of the interventions, there are improvements within the participants that are greater than the measurement error of 3.25 points known from the literature (48), this is a clear indication that the intervention influences motor function. However, if there is less or no change in the MDS-UPDRS-III but in one or more other parameters, this is still informative with regard to the effects of the different interventions and the underlying mechanisms. If no change is visible in any parameter under the influence of the interventions in any participant (through visual analysis and/or statistical tests), then the conclusion should be that there is no effect.

Visual inspection is the method of choice for single-subject research designs (SSRDs) (24). We will inspect, analyze, and describe the changes across the baseline and intervention phases (phase mean level and data spread), and we will compare the trend (slope) of scores with the baseline mean levels and variability (standard deviations) on outcomes for each intervention strategy and each patient. Longitudinal regression analysis with “phase” (i.e., condition) as the independent variable will be used for further confirmation (α = 0.05).

We will not explicitly test for group differences between patients in Hoehn–Yahr stage 1–2 or for patients in Hoehn–Yahr stage 2.5–3, but we will get insights on the direction of effects and effect sizes in the intervention phases compared with the baseline phases, which we will report descriptively for both participants in both subgroups.

The association between biomarkers and symptom changes will be quantified by Pearson correlation coefficients and corrected for age, between changes in BDNF, α-synuclein, and Nfl and change scores on primary and secondary parameters.

## Discussion

Accumulating scientific evidence supports the use of physical rehabilitation training and exercise as an effective non-pharmacologic therapy to improve motor symptoms, problems with mobility and gait (49, 50), and non-motor features, including mood, cognition, and sleep [for review, see Reynolds et al. (51)] for patients at all stages of PD. Intensive exercise may even induce neuroplastic disease-attenuating effects via endogenous production of neurotrophic factors, including BDNF (4, 5, 52, 53).

There has been considerable progress in the area of physical training-induced neuroplasticity effects in PD (11, 54, 55), though our understanding is far from complete. Studies reported that PD patient show increased serum NfL protein levels (17) compared to age-matched healthy controls. NfL is a marker for axonal damage and related to cortical degeneration in PD (15). As we have stated earlier, the effect of physical exercise training on NfL serum protein levels in early or late stage PD is currently unknown.

Neurotrophic factors, including BDNF, are soluble endogenously produced polypeptides that are involved in the development, growth, functioning, and regulation of neurons and neuron-supporting cells. Studies indicate that continuous high-intensity training, moderate-intensity CAE, or low-intensity exercise enhances serum and plasma BDNF levels (12, 22, 52, 56). A recent meta-analysis showed cumulative evidence of physical exercise induced improved serum BDNF protein levels in PD (11). It is plausible that besides activation of the neurotrophic pathway (10), physical training increases blood flux into basal ganglia (57) and that this increased flux may stimulate vascular endothelial cells to secrete BDNF as a response to blood shear stress in the vessels (58). BDNF also has known cytoprotective effects on the striatum (59).

Reduced aerobic and anaerobic capacity are common in the elderly and especially in PD patients, though the rate of capacity reduction can be decreased by adopting a more active physical lifestyle (1). The experimental intervention in this study, HIIT, has already been used in sports science to improve both aerobic and anaerobic capacity (18); and preliminary studies are emerging in the field of PD. It is plausible that HIIT can be a time-efficient type of exercise that integrates both aerobic and anaerobic exercise, thereby enhancing the motivation of PD patients to comply with the intervention and in the long run promote a more active lifestyle. To our knowledge, we are the first to explore the within-subjects responses of blood biomarkers with weekly repeated measurements over multiple weeks (during baseline, washout, and intervention periods) for the two different exercise conditions. The design of the study allows for careful individual exploration of the response patterns of different exercise strategies on both symptoms and biomarkers. The advantage is that a small sample is enough to get important new knowledge, which will facilitate future well-powered clinical trials.

This study primarily explores whether HIIT is more effective at improving motor and cognitive performance than moderate CAE. If our hypothesis that HIIT is more effective than moderate CAE is correct, future HIIT studies in PD patients can add other treatment types, such as cognitive stimulation. For example, the Park-in-Shape study (60) investigated the feasibility of adding “exergaming” elements to improve adherence. Furthermore, a recent publication of a large observational study in PD patients in the Netherlands highlights the cost-effectiveness of specialized goal-based physical activity therapy by reducing both health-care cost and disease-related complications (61), which should be explored in future studies by combining HIIT with exercise goals beyond mere completion of the protocol.

To increase the societal relevance and impact of the current study protocol, two Parkinson patients who were consulted during the design process of this study expressed optimism about the procedures presented in this protocol. They also acknowledged the need for a better understanding of individual neural response patterns to different exercise intensities.

## Data Availability Statement

The original contributions presented in the study are included in the article/supplementary material, further inquiries can be directed to the corresponding author/s.

## Ethics Statement

The studies involving human participants were reviewed and approved by Medical Ethics Committee of Amsterdam UMC, Vrije Universiteit Medical Center (nr. 2029.083). The participants provided their written informed consent.

## Author Contributions

EW, MH, WB, TV, MR, MN, and OH contributed to conception and design of the study. EW and MH wrote the first draft of the manuscript. EW, MH, OH, TV, and WB wrote sections of the manuscript. All authors contributed to manuscript revision, read, and approved the submitted version. On behalf of the HIIT-PD consortium members who contributed to topical discussions during the grant writing proces: H. W. Berendse, Y. D. van der Werf, D. Ferrazzoli, R. T. Jaspers, G. Kwakkel, V. de Groot.

## Conflict of Interest

The authors declare that the research was conducted in the absence of any commercial or financial relationships that could be construed as a potential conflict of interest.

## Consortium

H. W. Berendse^1^, Y. D. van der Werf^2^, D. Ferrazzoli^3^, R. T. Jaspers^4^, G. Kwakkel^5^, Y. Mahieu^5^, E. Goudzwaard^5^ and V. de Groot^5^

^1^Department of Neurology, Amsterdam Neuroscience, Amsterdam University Medical Center (UMC), Vrije Universiteit Amsterdam, Amsterdam, Netherlands

^2^Department of Anatomy & Neurosciences, Amsterdam Neuroscience, Amsterdam University Medical Center (UMC), Vrije Universiteit Amsterdam, Amsterdam, Netherlands

^3^Department of Parkinson's disease, Movement Disorders & Brain Injury Rehabilitation, Fresco Parkinson Center, ‘Moriggia-Pelascini’ Hospital - Gravedona ed Uniti, Como, Italy

^4^Laboratory for Myology, Department of Human Movement Sciences, Amsterdam Movement Sciences, Vrije Universiteit Amsterdam, Amsterdam, Netherlands

^5^Department of Rehabilitation Medicine, Amsterdam Movement Sciences, Amsterdam Neuroscience, Amsterdam University Medical Center (UMC), Vrije Universiteit Amsterdam, Amsterdam, Netherlands.
